# Negative Effects of SIGIRR on TRAF6 Ubiquitination in Acute Lung Injury In Vitro

**DOI:** 10.1155/2020/5097920

**Published:** 2020-10-15

**Authors:** Feng Tian, Qiang Lu, Jie Lei, Yunfeng Ni, Nianlin Xie, Daixing Zhong, Guang Yang, Shaokui Si, Tao Jiang

**Affiliations:** ^1^Department of Thoracic Surgery, Tangdu Hospital, Fourth Military Medical University, Xi'an, China; ^2^Department of Respiration, Third Hospital of Baoji, Baoji, China

## Abstract

In this study, the effects of single immunoglobin IL-1 receptor-related protein (SIGIRR) on tumor necrosis factor- (TNF-) receptor-associated factor 6 (TRAF6) ubiquitination in acute lung injury (ALI) were evaluated in both alveolar epithelial cells and alveolar macrophage cells in vitro. Our results found that SIGIRR negatively regulated TRAF6 ubiquitination and such SIGIRR inhibition could enhance the TRAF6 expression in both alveolar epithelial cells (AECs) and alveolar macrophage cells (AMCs). SIGIRR knockdown may increase NF-*κ*B activity via TRAF6 regulation by the classical but not the nonclassical NF-*κ*B signaling pathway. Such modulation between TRAF6 and SIGIRR could affect cytokine secretion and exacerbate the immune response; the IL-8, NFKB1, and NFKBIA mRNA levels were reduced after SIGIRR overexpression. The current study reveals the molecular mechanisms of the negative regulatory roles of SIGIRR on the innate immune response related to the LPS/TLR-4 signaling pathway and provides evidence for strategies to clinically treat inflammatory diseases.

## 1. Introduction

Acute lung injury (ALI) is an acute hypoxic respiratory dysfunction resulting from multiple factors [1–3]. The continuous progressive phase of ALI develops into acute respiratory distress syndrome (ARDS) [4, 5]. The pathological feature of the above process was the injury of alveolar capillary endothelial cells and alveolar epithelial cells (AECs) combined with extensive pulmonary edema and minimal atelectasis. ALI has a poor prognosis and can rapidly progress to multiple organ failures with a 30%~70% mortality rate [6–8]. Therefore, it is very important to study the pathogenesis and prevention measures of ALI. It has been proven that the lipopolysaccharide- (LPS-) Toll-like receptor-4 (TLR-4) signaling pathway plays a key role in the pathologic process of ALI [9–11]. Suppression of the LPS/TLR-4-mediated innate immune response is highly effective for the control of ALI.

In the development of ALI, TLRs play an important role in the above pathology process and regulate the innate immune response by inducing the release of various inflammatory factors [12, 13]. LPS/TLR-4 could modulate the above process by NF-*κ*B activation, leading to the release of a large number of cytokines and chemokines from AECs and alveolar macrophage cells (AMCs) [14, 15].

Single immunoglobin IL-1 receptor-related protein (SIGIRR), also named Toll-like receptor/interleukin-1 receptor 8 (TIR8), is a member of the TLR superfamily [16, 17]. It has been demonstrated that SIGIRR is highly expressed in epithelial tissues, including the kidney, colon, spleen, and liver, but is weakly expressed in the brain and muscle [18, 19]. In our previous study [20], it was first discovered that SIGIRR is highly expressed in human normal AECs obtained from 20 lung cancer patients with surgically resected adjacent lung normal tissues. Our data also provide evidence that SIGIRR overexpression could effectively inhibit LPS-induced ALI and inflammatory responses. The inflammatory response induced by LPS, IL-1, or other proinflammatory factors was more serious in SIGIRR knockout mice than in wild-type mice [21, 22]. In addition, some reports have shown that IL-1R/TLR-4 can directly interact with the intracellular TIR domain of SIGIRR [23, 24]. It has been shown that TLRs play a key role in the pathological process of ALI, and other studies have also proven that there are negative effects of SIGIRR on the TLR signaling pathway [25, 26], but the relationship between SIGIRR and lung injury and the detailed molecular mechanism are still unknown; in addition, there are few studies about the above process. Thus, exploring the downstream target molecule of SIGIRR and elucidating the mechanism of the above process will help us find new therapeutic methods for the clinical treatment of ALI.

It has been demonstrated that the downregulation of the LPS-TLR-4 pathway could significantly inhibit the severity of LPS-induced inflammation [27]. SIGIRR is one of the molecules that inhibits TLR-4. In previous reports, high expression of SIGIRR was observed in normal human AECs, and SIGIRR overexpression inhibited LPS-induced ALI [28, 29]. Tumor necrosis factor- (TNF-) receptor-associated factor 6 (TRAF6) can activate kinase 1 (TAK1) and TAK1-binding protein 2 (TAB2), which are related to NF-*κ*B activation and cytokine secretion [30, 31]. In addition, TRAF6 was the downstream target for IL-1R-associated kinase (IRAK) that could be recruited by MyD88. It has been reported that NF-*κ*B activation resulted from the TRAF6-TRIF-binding process, which could affect the immune response combined with TLR-4 [32–34].

However, it was still unclear if there is a relationship between SIGIRR and TRAF6 and if this interaction of the above two molecules would affect the downstream targets of the inflammatory response. Thus, in our current study, the interaction between SIGIRR and TRAF6 was explored by evaluating TRAF6 autoubiquitination regulated by SIGIRR expression. However, it is known that the activation of NF-*κ*B is related to the ubiquitination of TRAF6.

We hypothesized that such modulation between the above two molecules would change NF-*κ*B activation and cytokine secretion and affect the further immune response related to the effects of the LPS/TLR-4 signaling pathway. The implementation of the study will be helpful to reveal the molecular mechanisms of the negative regulatory roles of SIGIRR on the innate immune response and will provide experimental evidence for the clinical treatment of inflammatory diseases, especially for ALI.

## 2. Materials and Methods

### 2.1. Cell Culture

AECs and AMCs were obtained from the American Type Culture Collection (Manassas, VA). Cells were cultured with Dulbecco's modified Eagle's medium (DMEM, Gibco, Carlsbad, CA) supplemented with 10% fetal calf serum (FCS) and 1% penicillin/streptomycin at 37°C in a humidified incubator with 95% air and 5% CO_2_. Cells were passaged every 3 days to maintain growth. HEK293 cells were used and transfected with the indicated vectors in the glutathione S-transferase (GST) pull-down and coimmunoprecipitation experiments as previously described [35, 36]. In brief, the SIGIRR with HA and TRAF6 with Flag were prepared as previously described [20]. By immunoblotting analysis, SDS-PAGE methods were used to elute proteins. Anti-SIGIRR and anti-TRAF6 antibodies were used in the above protocol.

### 2.2. Western Blotting

First, lysing buffer including 150 mM NaCl, 0.01 M EDTA, 0.01 M EGTA, 0.01 M Tris-HCl (pH 7.5), 1% Triton X-100, 0.01 M *β*-glycerophosphate, 0.01 M Na_3_VO_4_, and 1 *μ*g/ml leupeptin was used to obtain the protein from AECs and AMCs. A BCA test was performed to determine the protein concentrations of the cell lysates. Then, the normal procedure of western blotting was performed as previously described [20]. Antibodies against TRAF6 (1 : 1000) and SIGIRR (1 : 1000) were purchased from Cell Signaling Technology (Beverly, MA, USA).

### 2.3. Quantitative PCR

Quantitative PCR, which was performed as previously described [20, 37], was used to observe the mRNA expression levels of TRAF6, SIGIRR, and the above cytokines.

### 2.4. NF-*κ*B Reporter Assay

After cell transfection, an NF-*κ*B firefly luciferase reporter was used to evaluate NF-*κ*B activity according to the manufacturer's instructions. First, the above cells were lysed in passive lysis buffer, and then, a dual-luciferase assay was used to quantify the luciferase activity [38, 39].

### 2.5. Statistics

Statistical analyses were performed using GraphPad Prism. All data are expressed as the mean ± SEM. *P* < 0.05 was considered statistically significant.

## 3. Results

### 3.1. Effect of SIGIRR on TRAF6 Ubiquitination

**Figure 1:**
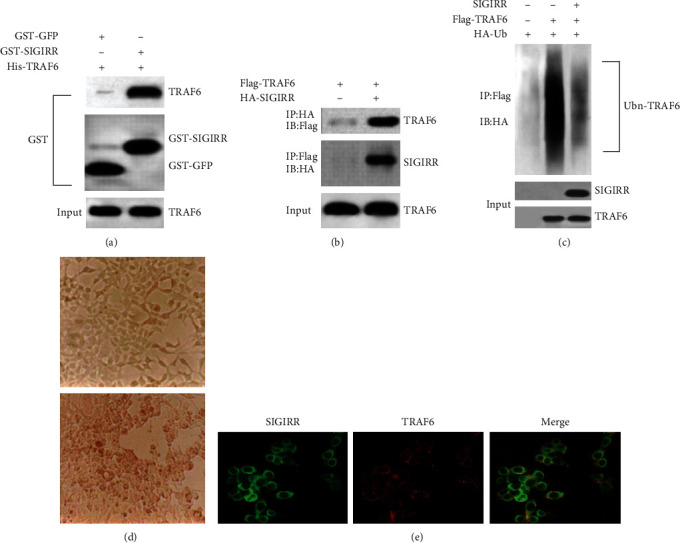
Analysis by pull-down and coimmunoprecipitation experiments of the interaction between SIGIRR and TRAF6 and resultant (auto) ubiquitinylation of TRAF6. (a) GST pull-down assay was performed using purified GST-fusion SIGIRR and His-tagged TRAF6 proteins. (b) A lysate from HEK293 cells cotransfected with Flag-TRAF6 or HA-SIGIRR was subjected to immunoprecipitation with anti-HA or anti-Flag antibody, respectively. (c) A lysate from HEK293 cells cotransfected with SIGIRR, Flag-TRAF6, or HA-ubiquitin, respectively, was subjected to immunoprecipitation with anti-Flag antibody. (d) HEK293 cells were cultured and cotransfected with the above regents. (e) Colocalization between SIGIRR and TRAF6 in HEK293 cells was also evaluated by immunofluorescence confocal microscopy.

The interaction between SIGIRR and TRAF6 was observed through GST pull-down and coimmunoprecipitation experiments using anti-SIGIRR and anti-TRAF6 antibodies. GST pull-down assay was performed using purified GST-fusion SIGGRR and His-tagged TRAF6 proteins. His-TRAF6 interacted with GST-SIGGRR, whereas GST alone did not exhibit any pull-down activity with His-TRAF6 protein. Lysed HEK293 cells including proteins were pulled down with purified GST-GFP or GST-SIGIRR (Figures 1(a) and 1(b)).

The results from the GST pull-down and coimmunoprecipitation experiments showed that there is an interaction between SIGIRR and TRAF6. HEK293 cells were cotransfected with SIGIRR, Flag-TRAF6, and HA-ubiquitin, and the results showed that the above cotransfection could reduce the TRAF6 ubiquitination level in HEK293 cells (Figure 1(c)). Ubiquitination modification could also regulate the following gene expression or function changes in the physiological processes. And thus, NF-*κ*B activation could be regulated by the ubiquitination of TRAF6 [40, 41]. HEK293 cells (Figure 1(d)) were cultured and transfected according to the previous protocol [35, 36]. In addition, colocalization between SIGIRR and TRAF6 in HEK293 cells was also evaluated by immunofluorescence confocal microscopy. The results suggested that SIGIRR and TRAF6 colocalized to the cytoplasm or the cytoplasmic membrane (Figure 1(e)).

### 3.2. Effect of SIGIRR on TRAF6 Expression Both in AECs and in AMCs

**Figure 2:**
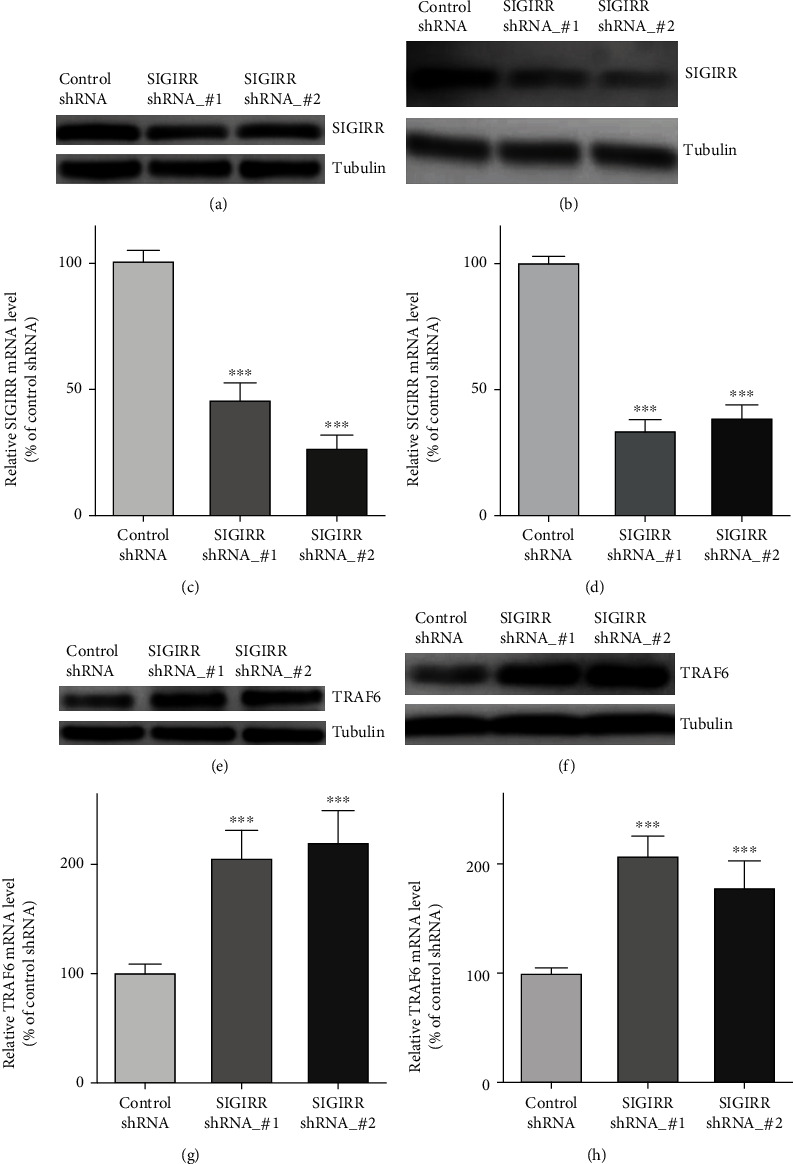
Effect of SIGIRR on TRAF6 expression both in AECs and in AMCs. (a, b) The results using western blot demonstrated that SIGIRR expression was reduced significantly both in AECs and in AMCs after transfection with SIGIRR_shRNA#1 or SIGIRR_shRNA#2. (c, d) The results using quantitative PCR demonstrated that SIGIRR mRNA levels were reduced significantly in AECs and AMCs after the above transfection. (e, f) TRAF6 expression by using western blot obviously increased in SIGIRR_shRNA-treated cells compared with those in control cells both in AECs and in AMCs. (g, h) TRAF6 mRNA levels by using quantitative PCR obviously increased in SIGIRR_shRNA-treated cells compared with those in control cells both in AECs and in AMCs. The data represent three independent experiments with four samples for each treatment. ^∗∗^*P* < 0.01 and ^∗∗∗^*P* < 0.001.

AECs and AMCs were first cultured by a normal protocol. To evaluate the effect of SIGIRR on TRAF6 expression, western blotting analyses and quantitative PCR assay were performed in the following experiments to detect TRAF6 expression and mRNA levels both in AECs and in AMCs, respectively. The results demonstrated that SIGIRR expression and the mRNA levels were reduced significantly both in AECs (Figures 2(a) and 2(c)) and in AMCs (Figures 2(b) and 2(d)) after transection with SIGIRR_shRNA#1 or SIGIRR_shRNA#2. After that, our results also provided clear evidence that both TRAF6 expressions (Figures 2(e) and 2(f)) and the mRNA levels (Figures 2(g) and 2(h)) increased obviously in cells that were treated with SIGIRR_shRNA compared with those in the untreated cells for both AEC and AMC groups.

**Figure 3:**
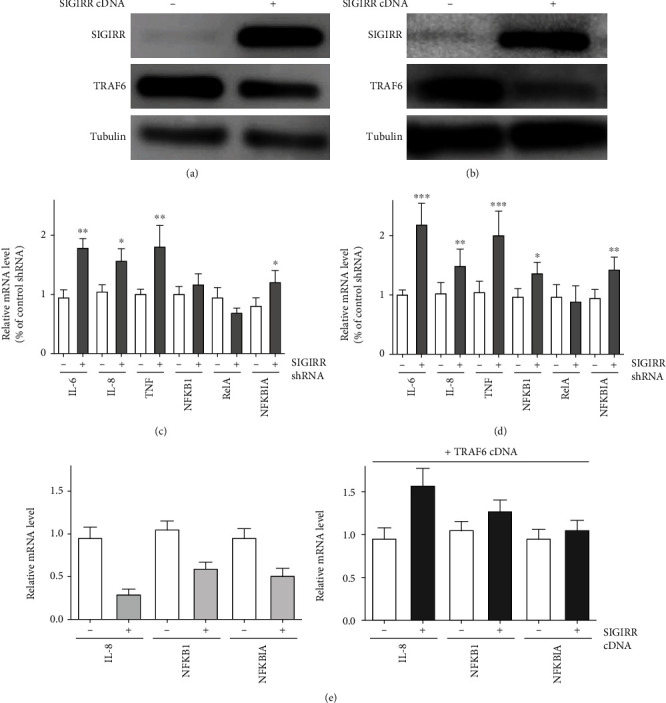
Effect of TRAF6 reversal of SIGIRR in AMCs. (a, b) SIGIRR overexpression was performed to detect changes on SIGIRR and TRAF6 in AECs and AMCs. (c, d) The mRNA levels of downstream NF-*κ*B signaling molecules, including IL-6, IL-8, TNF-*α*, NFKBIA, NFKB1, and RelA, were observed in the presence or absence of SIGIRR_shRNA in AECs and AMCs. (e) The rescue experiments were performed by cotransfection with TRAF6_cDNA and SIGIRR_shRNA in AMCs. The data represent three independent experiments with four samples for each treatment. ^∗∗^*P* < 0.01 and ^∗∗∗^*P* < 0.001.

Furthermore, SIGIRR_cDNA was transfected to obtain the overexpression of SIGIRR both in AECs and in AMCs. Using western blotting analyses, increased SIGIRR expression was observed after SIGIRR_cDNA transfection, and TRAF6 expression was decreased significantly both in AECs (Figure 3(a)) and in AMCs (Figure 3(b)) compared with that in control cells. Thus, the above data suggested that the negative regulation of SIGIRR on TRAF6 was observed and that such inhibition of SIGIRR by a specific shRNA could enhance TRAF6 expression both in AECs and in AMCs.

### 3.3. Effect of SIGIRR/TRAF6 on the NF-*κ*B Signaling Pathway Both in AECs and in AMCs

Next, the effect of SIGIRR expression on the NF-*κ*B target gene network was observed by quantitative PCR assay. After specific inhibition of SIGIRR expression by using SIGIRR_shRNA, the mRNA levels of several downstream NF-*κ*B signaling molecules, including IL-6, IL-8, TNF-*α*, and NFKBIA, were significantly increased both in AECs and in AMCs compared with the levels in the control group. However, for another two molecules, NFKB1 and RelA, the above significant enhancements in the mRNA levels were not obtained, and there were no changes in the presence or absence of SIGIRR_shRNA in AECs and AMCs. The difference was that IL-6, IL-8, TNF-*α*, and NFKBIA belong to the classical NF-*κ*B signaling pathway between the two above groups of downstream molecules, but NFKB1 and RelA were the molecules of the nonclassical pathway [31, 32]. The above results provide evidence that SIGIRR knockdown may affect the classical but not the nonclassical NF-*κ*B signaling pathway both in AECs (Figure 3(c)) and in AMCs (Figure 3(d)), but further details should be focused to clarify the underlying mechanism in the future. In addition, overexpression of SIGIRR was also performed to detect the changes on the above downstream molecules. The results showed that obvious inhibition of the IL-8, NFKB1, and NFKBIA mRNA levels in AMC was obtained after SIGIRR_cDNA transfection (Figure 3(e)).

Furthermore, the rescued experiments were also performed by cotransfection with TRAF6_cDNA in the presence or absence of SIGIRR overexpression in AMC, which reversed the effect of SIGIRR on NF-*κ*B downstream molecules. The IL-8, NFKB1, and NFKBIA mRNA levels were significantly enhanced after SIGIRR overexpression transfection in the presence of TRAF6_cDNA transfection in AMC (Figure 3(e)). Thus, the above data provide evidence that SIGIRR affects the downstream molecules of the classical NF-*κ*B signaling pathway by modulating TRAF6.

**Figure 4:**
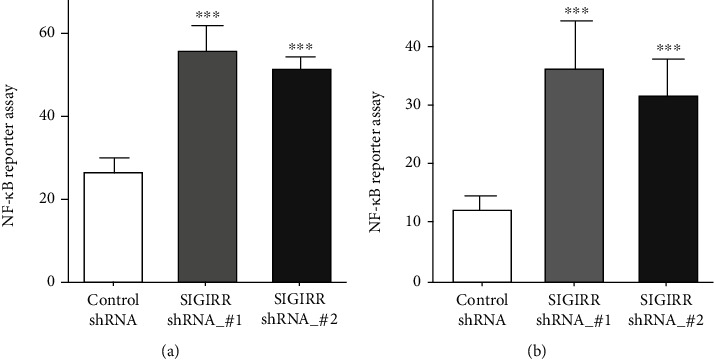
Inhibition of SIGIRR can affect the activity of the NF-*κ*B pathway in AECs and AMCs. (a, b) NF-*κ*B report experiments were performed to observe the effect of SIGIRR_shRNA on NF-*κ*B activity in AECs and AMCs. The data represent three independent experiments with four samples for each treatment. ^∗∗^*P* < 0.01 and ^∗∗∗^*P* < 0.001.

NF-*κ*B receptor experiments were performed to observe the effect of SIGIRR on NF-*κ*B activity. AECs and AMCs were transfected with SIGIRR_shRNA to reduce SIGIRR expression. The results of the NF-*κ*B activity assay showed that inhibition of SIGIRR expression significantly increased NF-*κ*B activity both in AECs and in AMCs (Figure 4).

### 3.4. Effect of SIGIRR on TNFAIP3 Expression Both in AECs and in AMCs

**Figure 5:**
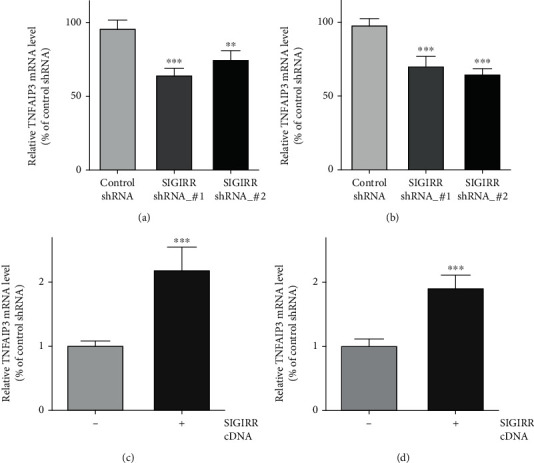
Effect of SIGIRR on TNFAIP3 expression both in AECs and in AMCs. (a, b) The TNFAIP3 mRNA levels both in AECs and in AMCs were evaluated after SIGIRR_shRNA transfection using the quantitative PCR assay. (c, d) TNFAIP3 mRNA levels were obviously enhanced both in AECs and in AMCs after SIGIRR overexpression by SIGIRR_cDNA application. The data represent two independent experiments with four mice per group. ^∗∗^*P* < 0.01 and ^∗∗∗^*P* < 0.001.

TNFAIP3 expression and the mRNA levels both in AECs and in AMCs were evaluated after SIGIRR_shRNA transfection using western blotting and quantitative PCR assay, respectively. It has been proven that NF-*κ*B activity was regulated by TNFAIP3 negatively [42–44]. The results showed that inhibition of SIGIRR expression could significantly reduce TNFAIP3 expression and the mRNA levels in the above cells compared with those in the control cells (Figures 5(a) and 5(b)). In addition, SIGIRR overexpression by SIGIRR_cDNA enhanced TNFAIP3 expression and the mRNA levels both in AECs (Figure 5(c)) and in AMCs (Figure 5(d)).

## 4. Discussion

ALI is a progressive, acute, hypoxic respiratory dysfunction that results from multiple factors [3, 4]. It has been demonstrated that suppression of LPS/TLR-4 greatly promotes the development of ALI. Inhibition of LPS/TLR-4 plays a key role in the above pathological process and mediates the innate immune response that is induced by different inflammatory factors [11–14].

In this study, we addressed the mechanisms through which the interaction of SIGIRR and TRAF6 affects ALI. The effect of SIGIRR on TRAF6 was observed by different methods. Our current study provided the following evidences: (1) SIGIRR and TRAF6 were colocalized in the cells, and overexpression of SIGIRR could reduce the ubiquitination level of TRAF6; (2) negative regulation of SIGIRR on TRAF6 was observed, and inhibition of SIGIRR could enhance the expression of TRAF6 both in AECs and in AMCs; (3) SIGIRR knockdown by shRNA may increase the NF-*κ*B activity by the classical NF-*κ*B signaling pathway but not the nonclassical pathway, and SIGIRR overexpression could reduce the IL-8, NFKB1, and NFKBIA mRNA levels, which could be rescued by TRAF6 overexpression; and (4) SIGIRR could affect TNFAIP3 expression.

SIGIRR, also named Toll-like receptor/interleukin-1 receptor 8 (TIR8), and a member of the TLR-IL-1R receptor superfamily were involved in the pathological process of ALI by regulating the NF-*κ*B activation to release the cytokines and chemokines, and increased SIGIRR expression could inhibit LPS-induced ALI by downregulating the LPS-TLR-4 pathway [17, 18]. MyD88 is involved in the pathway of IL-1R-associated kinase and TRAF6. In addition, SIGIRR could negatively regulate multiple LR-IL-1R receptor-mediated signaling transduction pathways, while the negative regulatory mechanism of SIGIRR has not been fully elucidated [23, 45]. Thus, the effect of the LPS/TLR-4 signaling pathway on the interaction of SIGIRR and TRAF6 was observed. The treatment of ALI by SIGIRR would be a possible route. However, whether the effect is related to its inhibition of TRAF6 further reveals the molecular mechanism by which SIGIRR negatively regulates inflammation. The results showed that SIGIRR and TRAF6 colocalize in cells in ALI. The ubiquitination of TRAF6 was reduced by SIGIRR overexpression, which regulates NF-*κ*B activation. Inhibition of SIGIRR significantly increased the expression of TRAF6 in alveolar epithelium and macrophages, thereby further affecting the activity of NF-*κ*B molecules and regulating the levels of the downstream signals IL-6, IL-8, TNF-*α*, and NFKBIA.

ALI and ARDS are clinically critical diseases. Currently, there are no specific therapies for ALI/ARDS [7]. TLRs are important pathogen pattern recognition receptors in the innate immune system [10]. The immune response combined with TLR-4 could induce the TRAF6-TRIF-binding process and NF-*κ*B activation [33]. Excessive activation of TLR signaling plays a key role in the uncontrolled inflammatory response regulated by SIGIRR. Taken together, the data presented herein provide a new perspective on the relevance of ALI. Thus, such modulation between TRAF6 and SIGIRR could affect cytokine secretion and induce a further immune response. SIGIRR plays a negative regulatory role in the above process, such as ALI, related to the LPS/TLR-4 signaling pathway. Our previous data provided the evidence that SIGIRR inhibits the transcriptional activity of NF-*κ*B and reduces the amount of cytokines produced, protecting these cells from acute LPS-induced damage [20]. In addition, TNFAIP3 could regulate NF-*κ*B activity negatively [43]. Current results also found that the TNFAIP3 level will be regulated by changing SIGIRR expression.

Our above results provided the data that SIGIRR could interact with TRAF6 and reduce its expression due to the ubiquitination that was the critical factor for NF-*κ*B activation. The current study revealed the molecular mechanisms of the negative regulatory roles of SIGIRR on the innate immune response and will provide experimental evidence for the clinical treatment of inflammatory diseases.

## Data Availability

The data used to support the findings of this study are included within the article.
